# The screening mammography paradox: better when found, perhaps better not to find

**DOI:** 10.1038/sj.bjc.6604349

**Published:** 2008-05-27

**Authors:** D A Berry

**Affiliations:** 1 Division of Quantitative Statistics, Department of Biostatistics, University of Texas MD Anderson Cancer Center, 1515 Holcombe Boulevard, Box 447, Houston, TX 77030, USA

In this issue, Wishart *et al* (2008) address whether ‘screen-detected breast cancer confers additional prognostic benefit to the patient, over and above that expected by any shift in stage at presentation’. Their conclusion is affirmative. Although this observation is not new and indeed is increasingly understood and accepted by the research community (Joensuu *et al*, 2004; Shen *et al*, 2005), their study is an important confirmation. One application of the observation has been in statistical models that make inferences about the relative contributions of screening and adjuvant therapy to the decrease in breast cancer mortality that has occurred over the last 20 years in the United States (and in other countries as well) (Berry *et al*, 2005, 2006b; Fryback *et al*, 2006). A conclusion of these models is that, taking therapy into account, the observed breast cancer incidence and mortality would be inconsistent unless some cancers preferentially detected by screening mammography had, in the terminology of one study (Fryback *et al*, 2006), ‘limited malignant potential’.

Wishart *et al* (2008) point out a clinical implication: the relatively good prognosis of screen-detected cancers means that clinicians are overtreating some patients. Indeed. The problem is that although we are learning rapidly about the biology of breast cancer and its impact on treatment (Bazell, 1998; Paik *et al*, 2004, 2006; Berry *et al*, 2006a; Hayes *et al*, 2007), our understanding is not yet sufficiently advanced to make clear which tumours need treatment, or which treatments are best for which biological subtypes. Nor do we know the proportion of patients in either detection category who will benefit from treatment. Knowing that fewer screen-detected than symptomatically detected tumours need treatment is not of much help unless we can identify which ones they are.

That screen-detected tumours have better prognoses is an important consideration in clinical research. Method of detection of breast cancer is not a standard prognostic factor recorded in clinical trials. This omission is regrettable. In some settings, method of detection may be as important as lymph node status in predicting disease recurrence. Happily, the research community is beginning to recognise this oversight. Trials such as TAILORx in the United States (Sparano, 2006; National Cancer Institute, 2008; Sparano and Paik, 2008) have incorporated method of detection on patient case report forms.

In their analyses of the importance of method of detection, Wishart *et al* (2008) correctly adjust for stage of disease and for the Nottingham Prognostic Index (NPI) more generally. The question of clinical interest is whether given the clinical, pathological, and demographic characteristics of the patient and her tumour, it helps to know the method of detection as well? Their answer is yes.

How can this be? It has always been clear that there is a stage shift associated with screening mammography: screening tends to find cancer earlier than otherwise. But it is surprising that a tumour clinically and pathologically identical to another tumour is fundamentally different, simply because of the way it was found. A possible explanation for the additional benefit of screen-detected tumours is a ‘within-stage shift.’ For example, some node-negative tumours harbour the potential to become metastatic (or already are metastatic!) while others do not. But as I discuss below, other explanations are possible.

The title of the Wishart *et al* (2008) article is ‘Screen-detected *vs* symptomatic breast cancer—is improved survival due to stage migration alone?’ This could be read as implying that screening improves survival. In the text they say, ‘Our results confirm a strong survival advantage of screening compared with symptomatic detection’. This is literally and incontrovertibly true. Their words will be seen by many readers to imply that screening mammography improves survival. But their study cannot address the question of screening effectiveness, nor do they claim to address this question.

There is an inherent aspect of the Wishart *et al* (2008) study that makes it difficult to state conclusions without having them misinterpreted. I speak from experience. There was substantial press coverage for my observation that method of detection is an important independent prognostic factor in breast cancer (Shen *et al*, 2005). However, despite my protestations, most reporters interpreted the study as meaning that screening was effective (which is the reason for the press coverage!). Perhaps my abilities to explain such matters are limited; after prolonged discussion with one reporter, she wrote an article with this headline in large lettering: ‘Mammograms Boost Survival Odds.’ In a nod towards accuracy, she added a subtitle, in much smaller lettering: ‘The screenings often detect slower-growing tumours, a new study finds.’

Wishart *et al* (2008) make clear that their observation is at least partially explained by lead-time and length biases. Lead-time bias is the easier to understand of these two biases. Suppose screening finds breast cancer an average of 3 years before it would become symptomatic. Then, this ‘lead time’ is added to the lives of women whose tumours are screen-detected and this additional 3 years may seem to be a benefit. But it is pure bias. Any benefit of screening has to be above and beyond this 3-year advantage. To get an unbiased estimate of screening effectiveness in a nonrandomised setting, one might subtract 3 years from the survival of every woman whose tumour is detected by screening. The problem is that in any particular setting we do not know that 3 years is the right adjustment: the true bias might be larger or smaller.

Length bias is more important than lead-time bias, at least in breast cancer. But neither its importance nor the concept itself is easy to understand. ‘Length’ refers to the tumour's presymptomatic period when the tumour is mammographically detectable. The length of this period is the tumour's sojourn time. Sojourn time varies from one tumour to another. (There is an obvious relationship between lead time and sojourn time; lead time is shorter because it requires actually finding the tumour during the presymptomatic period.) Sojourn time is typically positive, but it is negative for tumours that become symptomatic without being detectable on a mammogram. Breast tumours are heterogeneous, even after accounting for stage and other known clinical and biological characteristics. Aggressive tumours have shorter sojourn times because they grow faster. Indolent tumours have longer sojourn times. Screening finds tumours in proportion to their sojourn times, and therefore longer times and slower growing tumours are preferentially selected. This is length bias. (There are many analogues: when you look in the sky and see a shooting star, it is more likely to be one with a longer arc; when you reach into a newly opened bag of potato chips and select one, it is more likely to be big.)

A special case of length bias is overdiagnosis, when screening finds a tumour with a sojourn time so long that the tumour would not kill the woman even if it was never found.

Again, Wishart *et al* (2008) make clear that their observation is at least partially explained by lead-time and length biases. Their Figure 1 encompasses both, as they understand. Adjusting for NPI and other factors at least partly removes lead-time bias. But it does not remove length bias. Some tumours grow slowly. By their very nature such tumours have better prognoses than do rapidly growing tumours. Fast growing tumours will eventually become symptomatic, and among women who participate in screening mammography programs, they are likely to be the ones detected symptomatically between screenings (so-called interval cancers). Some tumours grow so slowly that they would never be detected were it not for screening. The consequence is overdiagnosis.

Therefore, although the authors are correct in worrying that screen-detected cancers may be overtreated, a greater concern is that some screen-detected cancers should never have been detected in the first place! The rub is that just as with treatment, we do not yet have a good understanding regarding which cancers we do not want to detect. Mammography is too crude a tool to make this distinction.
